# Green synthesis of silver nanoparticles through oil: Promoting full-thickness cutaneous wound healing in methicillin-resistant *Staphylococcus aureus* infections

**DOI:** 10.3389/fbioe.2022.856651

**Published:** 2022-08-23

**Authors:** Yuhan Wang, Qinmei Li, Xiaomin Peng, Zheng Li, Jun Xiang, Yunru Chen, Kaiyuan Hao, Shuaiyang Wang, Dongyang Nie, Yao Cui, Feifei Lv, Ying Wang, Wenda Wu, Dawei Guo, Hongbin Si

**Affiliations:** ^1^ State Key Laboratory for Conservation and Utilization of Subtropical Agro-bioresources, College of Animal Science and Technology, Guangxi University, Nanning, China; ^2^ Engineering Center of Innovative Veterinary Drugs, MOE Joint International Research Laboratory of Animal Health and Food Safety, College of Veterinary Medicine, Nanjing Agricultural University, Nanjing, China

**Keywords:** green synthesis, *L. cubeba* oil, AgNPs, antibacterial, multidrugresistance, wound infection

## Abstract

Due to the emergence of multi-drug resistant microorganisms, the development and discovery of alternative eco-friendly antimicrobial agents have become a top priority. In this study, a simple, novel, and valid green method was developed to synthesize *Litsea cubeba* essential oil-silver nanoparticles (Lceo-AgNPs) using Lceo as a reducing and capping agent. The maximum UV absorbance of Lceo-AgNPs appeared at 423 nm and the size was 5–15 nm through transmission electron microscopy result. The results of Fourier transform infrared and DLS showed that Lceo provided sufficient chemical bonds for Lceo-AgNPs to reinforce its stability and dispersion. The *in vitro* antibacterial effects of Lceo-AgNPs against microbial susceptible multidrug-resistant *Escherichia coli (E. coli)* and *methicillin-resistant Staphylococcus aureus (MRSA)* were determined. The minimum inhibitory concentration (MIC) and minimum bactericidal concentration (MBC) of Lceo-AgNPs against *E. coli* were 25 and 50 μg/ml. The MIC and MBC of Lceo-AgNPs against MRSA were 50 and 100 μg/ml, respectively. The results of scanning electron microscopy showed that the amount of bacteria obviously decreased and the bacteria cells were destroyed by Lceo-AgNPs. *In vivo* research disclosed significant wound healing and re-epithelialization effects in the Lceo-AgNPs group compared with the self-healing group and the healing activity was better than in the sulfadiazine silver group. In this experiment, Lceo-AgNPs has been shown to have effects on killing multidrug-resistant bacteria and promoting wound healing. This study suggested Lceo-AgNPs as an excellent new-type drug for wound treatment infected with multidrug-resistant bacteria, and now expects to proceed with clinical research.

## 1 Introduction

Skin is the largest organ of the human body. As the first line of defense, skin injury can be easily infected by microbes, extending the healing process and inducing serious pain, even threatening life. One of the big challenges in the pharmaceutical and biomedical fields is resistance to pathogens or bacteria in humans and animals (Huang et al., 2017; Bellu et al., 2021). In this context, antibiotic resistance is currently causing widespread concerns about the emergence and re-emergence of multidrug-resistant (MDR) pathogens (Pungle et al., 2021). In recent years, the situation has become further complicated by emerging antibiotic resistance (Baptista et al., 2018; Mousavi et al., 2018). Methicillin-resistant *Staphylococcus aureus* (*MRSA*) is a typical example, which has become a most widespread pathogen in clinical tissue infections and critically threatens public health. In the past decades, an increasing number of resistance genes have been discovered in *E. coli*, most of which appeared through horizontal gene transfer. To acquire resistance genes from other bacteria or transfer resistance genes to others, *E. coli* can act as a donor and recipient in an entrepreneurial gene pool. In general, antimicrobial-resistant *E. coli* has been listed as a major challenge for humans and animals around the world and needs to be seen as a real public health concern (Banin et al., 2017; Poirel et al., 2018). It is of great significance for treatment of infectious wounds to promote wound healing while effectively inhibiting the growth of antibiotic-resistant bacteria.

To sidestep this problem, as a new antimicrobial agent with broad-spectrum antibacterial effects, AgNPs have been extremely desired to replace antibiotics with no effects and combat infections caused by multidrug-resistant bacteria (Miyoshi et al., 2010; Liao et al., 2019). Although the bactericidal mechanism of AgNPs is not very clear, some studies have discussed the mechanism according to their research results. Subsequently, AgNPs may infiltrate bacterial cell walls and regulate cell signaling by dephosphorylation of peptide substrates on tyrosine residues. Otherwise, the antibacterial properties of AgNPs depend on the size, morphology, stability, and (chemical and physical) properties of the nanoparticles (Zhang et al., 2008).

In general, AgNPs can be utilized after purification, but the difficulty and cost of removing harmful compounds limit the widespread use of AgNPs (Luo et al., 2015). To solve this problem, green-synthesis method was developed to synthesize more economic AgNPs (Kim et al., 2015; Kim et al., 2019). The phyto-mediated synthesis is an efficient, favorable, and most acceptable biosynthetic method for the synthesis of metal nanoparticles, which stand out for their inherent biocompatibility and rich availability. At present, various plant parts such as bark, leaf, fruit, stem, and seed extracts have been successfully utilized in the synthesis of silver nanoparticles. A previous study reported that AgNPs produced in a green-synthesis way had better biological activity due to the presence of plant derivatives (Marslin et al., 2018). In this way, the quick and high concentration of green-synthesis has significance for nanotechnology with more and more requirements.

Essential oil is the blend of volatile organic compounds in plants. Many studies have demonstrated that the secondary metabolites present in plant essential oils are the main composition of AgNPs (Vidya et al., 2014). For example, alcohol, esters, ethers, aldehydes, ketones, lactones, phenols, and phenol act as surface active molecules to stabilize nanoparticles (Song and Kim, 2009). Many plant compounds, such as citral, limonene, melatonin, coriander alcohol, geraniol, vanillin, and camphene, are present in *L. cubeba* (Mothana et al., 2016). Nonetheless, due to the irritation to mucosa and the limitation in storage, Lceo has a great limitation in clinical application (Xia et al., 2021). However, the Lceo capping to AgNPs for detecting antibacterial ability has not been researched. Therefore, in this study, quick and valid synthesis of AgNPs using Lceo was detailed, and the antimicrobial effect of synthesized Lceo-AgNPs was also detected. In this way, Lceo can be applied to clinical treatment in a better way.

The green synthesis of AgNPs as an original material has been a new trend relying on the characteristics of a safe reaction process and tremendous potential for antibacterial effects. To date, different plant extracts have been applied for synthesis of AgNPs, but plant essential oils are barely used in green synthesis of AgNPs. Lceo as a natural plant oil with abundant bonds was employed to synthesize AgNPs in green-synthesis way (Sendova et al., 2006; Yousaf et al., 2020). The aim of this study was to exploit Lceo-AgNPs in a green-synthesis way and replace antibiotics for wound multidrug-resistant bacteria infection. On the one hand, it postponed and decreased the possibility of antibiotic-resistant microbial. On the other hand, it also expanded the application scope of Lceo.

Therefore, in this study, we utilized Lceo as a reducing agent of AgNO_3_ to synthesize low-toxicity AgNPs with tiny particles, strong dispersion, and stability in a green-synthesis way. Meanwhile, Lceo-AgNPs exerted antibacterial activity on antibiotic-resistant *E. coli* and *MRSA*. In order to study the effects on wound healing and apply Lceo-AgNPs to clinical research, glycerin and gelatin were treated as carriers of Lceo-AgNPs and applied to full-thickness wound of mice infected by *MRSA*, increasing treatment efficiency and alleviating the pressure produced by bacteria antibiotic resistance.

## 2 Materials and methods

All chemicals and reagents used in this study were of analytical grade. Silver nitrate (AgNO_3_; Solarbio) and cell culture media (LB; 1640) were obtained from commercial sources. The PBS, starch broth, 1640 broth, and AgNPs were purchased from Beijing Solarbio Science & Technology Co., Ltd. (Beijing, China). Multidrug-resistant microbial strains containing *E. coli* named *EC14* and *MRSA* were obtained from The First People’s Hospital of Nanning (Guangxi Province, China) and identified and stored in the laboratory of Chinese veterinary medicine of Guangxi University (China).

## 3 Green-route synthesis of AGNPS

The chemicals, such as Lceo and AgNO_3_, were obtained from Jiangxi Cedar Natural Medicinal Oil Co., Ltd. (Jiangxi, China), and Sinopharm Chemical Reagent Co., Ltd. (Shanghai, China), respectively. Tween-80, anhydrous ethanol, and NaOH of AR were acquired from Sinopharm Chemical Reagent Co., Ltd. (Shanghai, China) and were used as obtained without further purification. All the microbial culture media were acquired from Beijing Aoboxing Biology Technology Co., Ltd. (Beijing, China). According to the previous experiment of our research team (Chen et al., 2021), Lceo was emulsified using tween-80 and anhydrous at 1:4:2 and diluted into 0.01 g/ml. A volume of 1 ml AgNO_3_ (0.1 M) was added to the Lceo after emulsion in different volumes with pH = 9 at 50°C in ultrasound (250 W) for 6 h (Nour El Din et al., 2016; Wang et al., 2018). To detect the best synthesis ratio, the effect of the amount of Lceo was examined at increasing ratios from 1:1 to 5:1 of Lceo and AgNO_3_ (Smekalova et al., 2016).

### 3.1 Characterization of Lceo-AgNPs

We used a UV-vis spectrophotometer (NanoDrop 2000 C, Thermo Scientific, United States) to scan Lceo-AgNPs mixture solution in the rage of 300–600 nm at a resolution of 1 nm. We added 1–2 μl Lceo-AgNPs solution to the probe of ultra-micro spectrophotometer and performed the scan within the range of 300–600 nm (Karuppaiah et al., 2020).

In order to estimate the particle size distribution and zeta potential, we measured a colloidal silver nanoparticle solution at 30 and 90°C detection angle with a Nanosizer (Nano-ZS90X, UK). The prepared Lceo-AgNPs solution was diluted and dropped into a plastic colorimetric dish with a dropper. The particle size distribution was measured by a series of potentiometers. The effective electric charges on the surface of nanoparticles were measured by zeta potential, which played a key role in determining the stability of the aqueous suspension system of nanoparticles (Yang et al., 2015).

The prepared Lceo-AgNPs were centrifuged at 12,000 rpm for 90 min in a high-speed centrifuge, the supernatant was discarded, and the precipitate was collected. The precipitate was mixed with deionized water and centrifuged again, and repeated three times to remove the other residual substances. The collected precipitate was placed in a glass dish, sealed with plastic wrap, and poked with a toothpick through several small holes. The samples were put in the freeze-dryer and the liquid samples were completely dried into powdered samples, which were obtained as solid samples of Lceo-AgNPs; 3–10 mg of Lceo-AgNPs powder was loaded into the sample tube, and the morphology and particle size of the Lceo-AgNPs particles were observed by transmission electron microscopy (TEM) (JEM-2100, Japan Electric Technology Co., Ltd., Japan). Test conditions: the acceleration voltage was 200 kV, the point resolution was 0.24 mm, and the line resolution was 0.14 nm (Kanwal et al., 2019).

Fourier transform infrared (FTIR) is an important characterization method commonly used in the study of plant synthesis of nanomaterials. Through the characterization and comparison of the functional groups contained in plant and materials, basic information about the compounds involved in synthesis was obtained. The FTIR spectro-photometer (Bruker Optics, Shanghai, China) was recorded between 4,000 and 400 cm^−1^ to clarify the composition of chemical bonds of Lceo and Lceo-AgNPs (Liu et al., 2018).

XRD was utilized to analyze the crystal structure of a substance to test whether the product obtained in this study was the AgNPs crystal. The dried Lceo-AgNPs powder sample was used for the detection. The voltage was set to 40 kv, the current to 40 mA, *λ* = 0.303 nm, the scan rate to 6°/min, and the scan range to 10°–90°.

The composed Lceo-AgNPs were collected after centrifugation at 13,000 rpm for 1 h to remove redundant chemicals and washed three times with deionized water, then dried using freeze drier (LC-10N-50D, Shanghai Lichen Bangxi Instrument Technology Co., Ltd. China). The collected Lceo-AgNPs were used for further characterization analysis and antibacterial experiments.

### 3.2 Antibacterial assay

#### 3.2.1 Antibacterial susceptibility

To explore multidrug-resistant activity of *EC 14*, rifampicin, tetracycline, meropenem, colistin, fossils, penicillin, amikacin, and ampicillin purchased from Beijing Solarbio Science & Technology Co., Ltd. (Beijing, China) were used.

The antibacterial effect of Lceo-AgNPs was assessed against multidrug-resistant *E. coli* and *MRSA*. The broth dilution technique was used to detect minimum inhibitory concentration (MIC) and minimum bactericidal concentration (MBC), which is consistent with the European Committee on Antimicrobial Susceptibility Testing (The European Committee on Antimicrobial Susceptibility Testing, 2020). The bacteriostatic rate of bacteria incubated for 24 h at 37°C was shown at 99.9%, which is defined as MIC. To examine the amounts of microbes, a small part of solution from each well was transferred to agar plates at 37°C and incubated for 24 h, and the smallest concentration with no visible bacteria growth seemed as MBC. The analysis of MIC was conducted on a 96-well plate to analyze the antibiotic resistance of *E. coli* strains and the antibacterial effect of Lceo-AgNPs against these strains. The bacteria suspensions including *E. coli* and *MRSA* were diluted into 10^5^ CFU/ml with MHB broth and 50 μl mixtures of bacteria were added into each well of 96-well microplate. Subsequently, various concentrations of Lceo-AgNPs were added into each well. After that, 96-well plates were cultured at 37°C for 24 h and recorded the results. Next, 50 μl solutions were sucked out from each well without visible bacteria into LB agar and cultured at 37°C for 24 h to examine the MBC. All the experiments above were repeated three times.

#### 3.2.2 Time-killing curve test on antibiotic-resistant *E. coli* and *MRSA* treated with Lceo-AgNPs

The method was based on previous experiments with some modifications (Xiao et al., 2015; Hakeem et al., 2019). The time-killing curve test was conducted to verify the time it took to kill all the bacteria. The *EC 14* and *MRSA* were cultured in LB broth at 37°C to 10^7^ cfu/ml. Lceo-AgNPs were added to each tube, whose concentrations were adjusted into MIC, MBC, respectively, and cultured at 37°C. The LB broth tube-added equal sterile saline was set up as the controls. After *EC 14* solution was incubated for 15, 30, 60, and 120 min and MRSA were incubated for 60, 120, 240, and 360 min, 100 μl of the mixture was diluted into different concentrations with sterile saline and added to LB agar plates. The bacterial solution was evenly coated on the plates with ss-spreaders and incubated at 37°C for 18 h. The number of colonies (M) was selected to be in the range of 30–300 dilutions (N), and the bacterial concentration of the original bacterial solution was calculated according to the formula. All tests were repeated three times, and the average value was taken as the final result and recorded.

$$Lg^{\left(\mathrm{CFU/mL}\right)}=N\times Lg^{\;\left(10\times M\right)}$$

N: dilution multiple.

M: colony count (30–300) on LB plate.

#### 3.2.3 Scanning electron microscopy analysis on the *E. coli* disposed by Lceo-AgNPs

The morphological features of *EC14* and *MRSA* were revealed by scanning electron microscopy (SEM) (GP 2008, Servicebio). The bacteria were cultured in 10^7^ cfu/ml of MHB broth. The *EC 14* and *MRSA* solutions were treated with Lceo-AgNPs of MBC cultured for 2 h. Then, the sediments were washed with PBS three times and gathered after centrifugation at 4,000 rpm for 10 min, which were fixed with the 2.5% glutaraldehyde. After washing with PBS, distilled water was mixed with bacteria solutions, then a drop of the mixture was added on the coverslip with a dropper. After being adsorbed for 2 min, the filter paper was used to aspirate excess solution. This coverslip was attached to the inner wall of the cap of the brown bottle with double-sided tape and fixed with 1% osmium acid for 2 h. Then, the coverslip was attached to the sample table and observed after gold spraying (Zengin and Baysal, 2014). The bacteria treated with saline were set as controls.

### 3.3 Cytotoxicity assay

The method was based on previous experiments (Paul et al., 2015; Nguyen et al., 2016); 1 ml starch broth was injected into the peritoneal of mice to trigger an inflammatory reaction and promote the proliferation of macrophages. Sterile PBS was injected into the peritoneal after 48 h and flushed out the macrophages which were diluted and cultured in 1640 broth supplemented with 10% fetal bovine serum (FBS), 100 U/ml penicillin, and 100 μg/ml streptomycin at 37°C in a humidified atmosphere with 5% CO_2_; 100 μl cell suspension and different concentrations of Lceo-AgNPs and AgNPs were seeded into 96-well plates at a density of 2 × 10^5^ cell/well and cultured at 37°C in a humidified atmosphere with 5% CO_2_ for 24 h. Cell viability was assessed using the MTT assay according to previous studies and made some changes. Briefly, 20 μl MTT (5 mg/ml) was added into each well except for control samples which were treated without AgNPs. Following an additional 4 h of incubation at 37°C, the culture medium was removed and replaced with DMSO (150 μl/well). Absorbance was measured at 570 nm using an enzyme-standard instrument. The same concentrations of AgNPs were used as positive controls (McConville et al., 2022). All the experiments mentioned above were repeated three times. The cell viability percentage was calculated as follows:

$$\left(OD_{\mathrm{of Lceo-AgNPs}\;\mathrm{treated sample}}/OD_{\mathrm{of control sample}}\right)\;\times 100\%.$$

## 4 *In vivo* study

### 4.1 Ointment formulation

On the basis of previous study, preparing glycerin–gelatin paste loaded with Lceo-AgNPs could take full advantage in wound healing through its humidity retention and absorbability (Zhao et al., 2017). 50% glycerin, 20% gelatin, and deionized water were mixed with a volume ratio of 1:7:2 and then boiled to evaporate water. The yellowish and thick paste was finally prepared and blended fully with Lceo-AgNPs and the final concentration was 100 μg/ml.

### 4.2 *In vivo* anti-infection and topical treatment

This method was based on previous experiments and made some difference (Krishnan et al., 2018; Montaser et al., 2021). 60 healthy male KM mice, weighing 18–22 g, were provided by Changsha Tianqin Biotechnology Co., Ltd. Silver sulfadiazine was purchased from Guangdong Hengjian Pharmaceutical Co., Ltd. All experimental procedures were approved by the Animal Research Ethics Committee of Guangxi University, Nanning, China (GXU-2021-164). The full-thickness cutaneous wound model infected by the *MRSA* was used to evaluate *in vivo* anti-infection effect and wound-healing activity. Routinely fed mice were generally anesthetized by intraperitoneal injection of 0.1 mg/ml chloral hydrate (0.3 ml/100 g). Then, full thickness wounds (8 mm in diameter) were produced on the dorsal area of each mouse which had already been shaved and sterilized with 75% ethanol. To establish antibiotic-resistant infection, 100 μl *MRSA* suspension (10^7^ CFU/ml) was inoculated in wounds for 1 day. All mice with infective wounds were randomly assigned into three groups (B: self-healing group, C: Lceo-AgNPs group, and D: silver sulfadiazine group). The negative control group (A) was filled with healthy mice.

After *MRSA* infection for 24 h, the Lceo-AgNPs ointment and silver sulfadiazine were applied to the infective wound once a day until the wound healed. The wound areas were monitored every alternate day.

Five mice were sacrificed after they healed for 0, 4, and 14 days. The wound/healthy skins at the wound sites of three mice of each group were collected after healed for 4 and 14 days to prepare paraffin sections for H&E staining test. The samples were fixed in 4% paraformaldehyde for 24 h, then dehydrated in gradient alcohol, transparent in xylene, wax dipped, embedded in paraffin, and finally sectioned. The sections were dewaxed in the xylene dewaxing cylinder for 10 min, and then rehydrated in gradient alcohol for 5 min. After being washed off the alcohol, the sections were dipped in hematoxylin for 3 min and separated by 1% hydrochloric acid, rechromatized in ammonia. The nuclei were stained in eosin solution for 1 min after microscopic observation. The sections were dehydrated in 100% alcohol, sealed with neutral resin for microscopic observation after being rinsed in xylene and observed under the microscope (Orlowski et al., 2020).

The wound areas were recorded and photographs were taken on days 4, 7, and 14, respectively. Wound contraction (%) was calculated following the design formulas:

$$\mathrm{Wound}\;\mathrm{healing rate=}\left(A_{0}-A_{t}\right)/A_{0}\times 100\%.$$

A_0_: wound areas on day 0 of healing administration;

A_t_: wound areas on day t of healing administration.

Blood samples of mice in each group were collected on days 0, 4, and 14 after treatment for ELISA containing C-reactive protein (CRP) and I collagen (Guangxi research assistant Gene Technology Co., Ltd.).

### 4.3 Statistical analysis

The results of characterization of Lceo-AgNPs, cell viability, time-killing curve, and wound-healing rate were analyzed in GraphPad Prism 8.0.1 (GraphPad Software Inc., San Diego, CA, United States). The ANOVA and Turkey Duncan multiple range tests of IBM SPSS Statistics 20 were utilized to perform statistical analysis. *p* < 0.05 was considered statistically significant. The results of all the experiments were expressed as the mean value of three independent replicates ± standard deviation (SD). The result of immunohistochemistry was expressed as SD ± standard error (SE).

## 5 Results and discussion

### 5.1 Synthesis of Lceo-AgNPs

**FIGURE 1:**
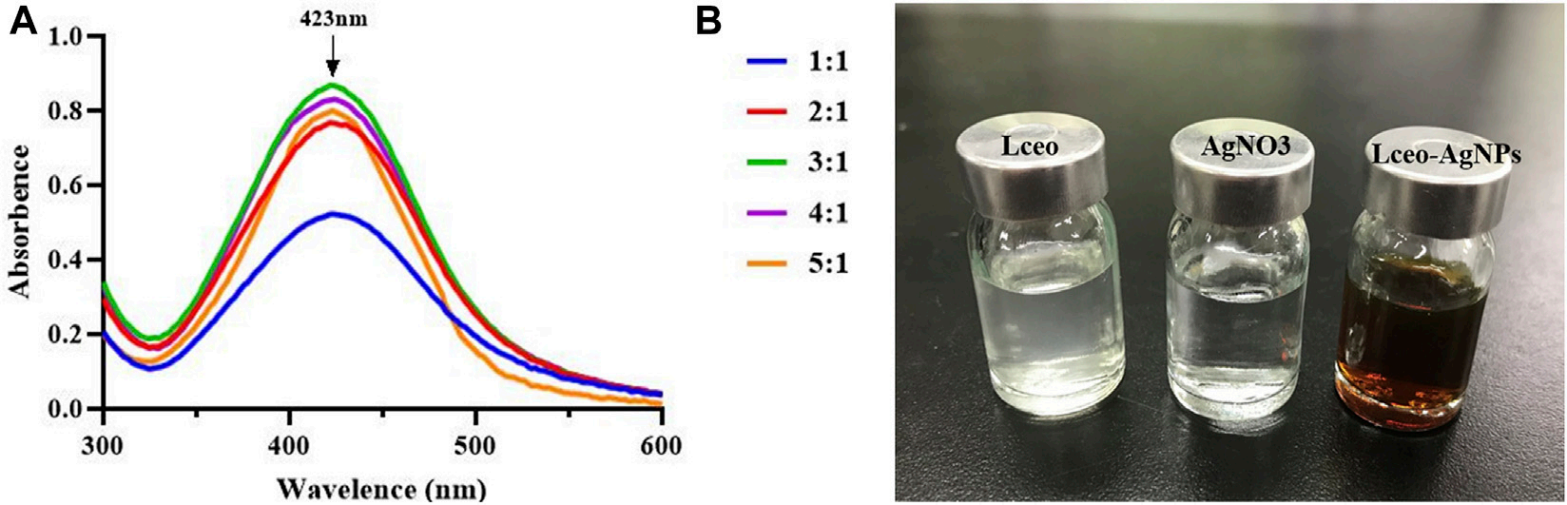
UV-vis wavelength spectra of Lceo-AgNPs with different ratio of Lceo: AgNO_3_ in the range of 300–600 nm **(A)** and the colors of Lceo, AgNO_3_, and Lceo-AgNPs **(B)**.

This study aimed to produce AgNPs with a direct molecular mechanism, utilizing Lceo as a strong reducing and stabilizing agent. Therefore, this producing way of AgNPs was entirely based on the green-synthesis route. Green-synthesis of AgNPs was indicated by gradual color development in the reaction solution. The color of the reaction solution was changed as soon as 0.1 M AgNO_3_ was added into Lceo and gradually changed into deep brown, which meant the synthesis of AgNPs was fast. In fact, the color changes into brown occur due to the excitation of surface plastic vibrations of the AgNPs. UV-vis was used to confirm the synthesis of Lceo-AgNPs. AgNPs have a specific maximum absorption peak between 420 and 450 nm. Figure 1A shows the UV-vis of Lceo-AgNPs synthesized by different ratios of Lceo and AgNO_3_. Lceo-AgNPs exhibited highly stable and the highest surface plasma resonance (SPR) band at 423 nm while the ratio of Lceo and AgNO_3_ was 3:1, symbolizing the highest concentration of synthesized AgNPs, which may have been due to Lceo being absolutely reacted with AgNO_3_ in 3:1. When the ratio were lower than 3:1, AgNO_3_ could not be reduced completely. However, when the ratio were higher than 3:1, the resultant quantity of Lceo-AgNPs was lower. As shown in Figure 1B, compared with neutral Lceo and AgNO_3_, AgNPs with the ratio of 3:1 was changed into deep brown after 6 h with 250 W ultrasonic. These results indicated that Lceo had significant potential for preparing AgNPs in the green-synthesis route. The Lceo-AgPNs were prepared with ratio of 3:1 in 50°C with 250 W ultrasonic for 6 h to proceed with characterization experiments.

### 5.2 Characterization of Lceo-AgNPs

#### 5.2.1 DLS analysis

**FIGURE 2:**
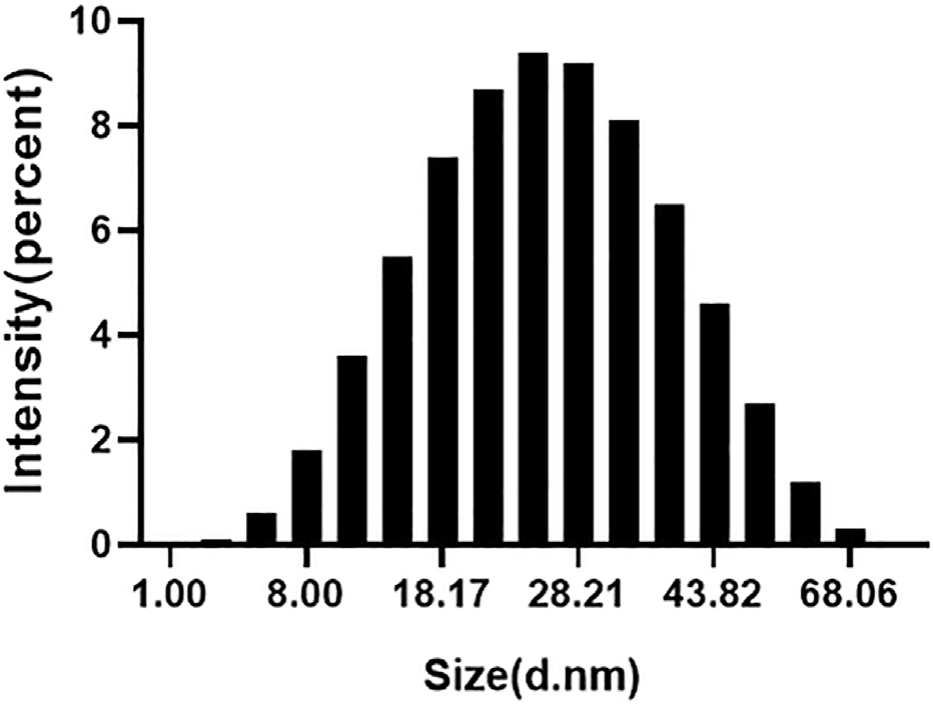
Nanosizer of as-prepared Lceo-AgNPs.

DLS analysis was performed to determine the average size and zeta potential of silver nanoparticles. As shown in Figure 2, the size of the synthesized nanoparticles varies from 5 to 70 nm. The results showed that the average particle size of the nanoparticles was 37.87 ± 0.2 nm. The particle size displayed by DLS was different from the TEM result, which was due to the chemical counting of the larger particle size of other unreacted components in Lceo.

**FIGURE 3:**
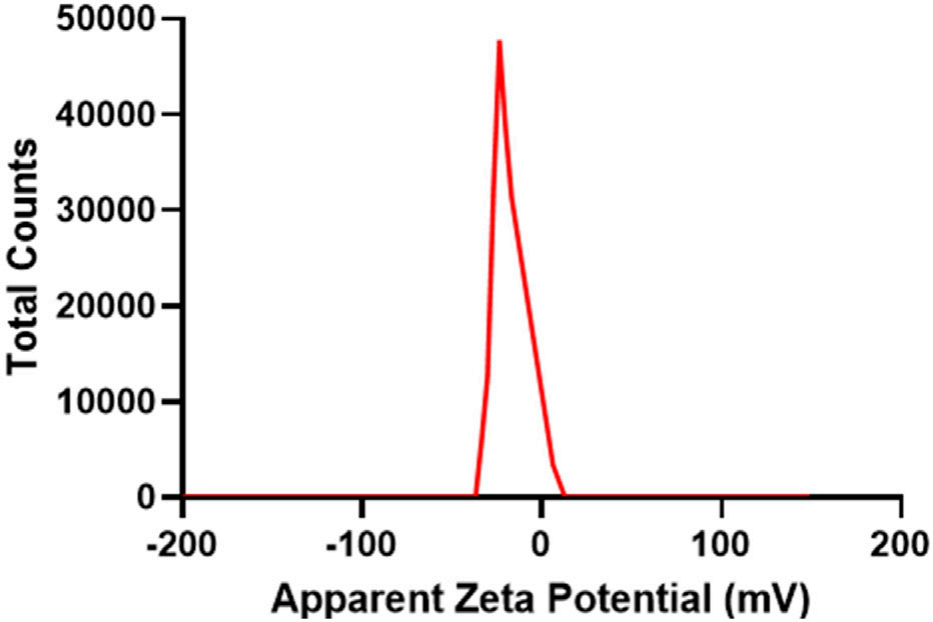
Zeta potential of as-prepared Lceo-AgNPs.

The zeta potential (Figure 3) of Lceo-AgNPs was −36.8∼12.9, which indicated that AgNPs reduced by Lceo through the green-synthesis route were stable enough and significant dispersion to retain splendid biochemical effects. This was due to the fact that biomolecules bound to the AgNPs surface and substantially expanded its surface charge, thus enhancing its stability by inhibiting aggregation.

#### 5.2.2 FTIR analysis

**FIGURE 4:**
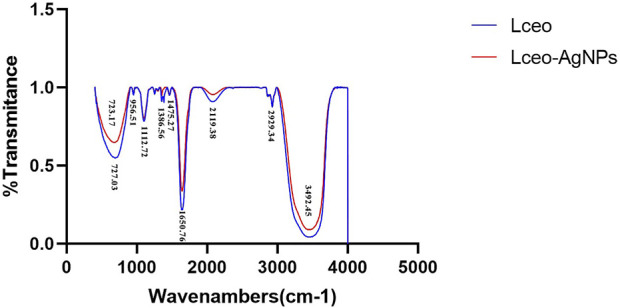
FTIR absorption spectra of Lceo-AgNPs and Lceo.

The result of FTIR (Figure 4) proved that the possible bioactive molecules and capping agents were responsible for promoting the generation of Lceo-AgNPs and improving the stability to maintain biochemical properties. The same vibration with decreasing intensity and movement of functional groups vibration were objected to Lceo-AgNPs solution, which indicated the surface functionalized phytochemicals of Lceo-AgNPs. The strong peaks in 723.17 and 727.03 represented aromatic hydrocarbons with C-H stretching vibration. The strong bands in 956.51, 1386.56 and 1475.27, 1650.76, 2929.34, and 3492.45 corresponded with C=C or N-O, -C-C, C=O, H-C-H, and O-H stretching vibration, respectively. The strong speaks at 2119.38 and 1112.72 resulted from the alkyne and ether in aliphatic series or ring (Khan et al., 2017). Hence, we concluded that the potential biomolecules in Lceo contained a mixture of carbonyl compounds. The citral, limonene, linalyl isobutyrate, α-vinyl acetate, geraniol, and other organic substances in Lceo endowed silver nanoparticles with rich functional groups to maintain the stability of silver nanoparticles.

#### 5.2.3 TEM

**FIGURE 5:**
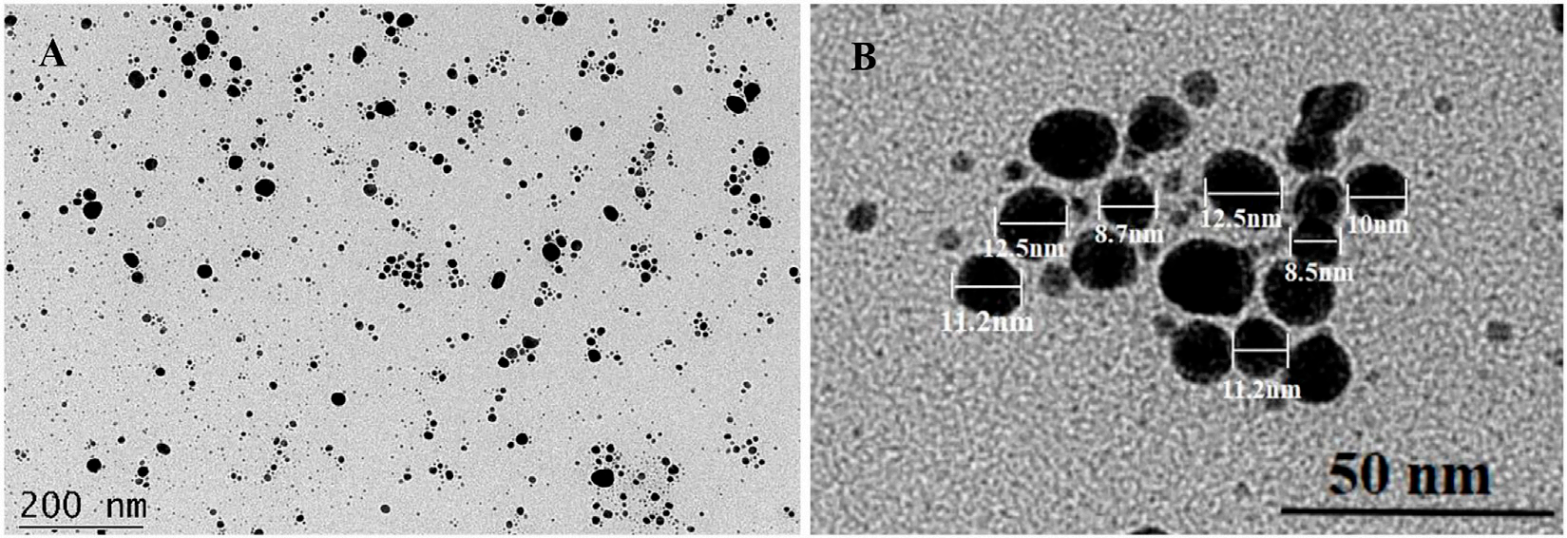
TEM images of dispersed Lceo-AgNPs.

TEM was used to confirm the shape and size of Lceo-AgNPs as shown in Figure 5. TEM analysis of Lceo-AgNPs showed that the particle size was small and the shape was uniformly spherical. In addition, a small number of large particles were found in Figure 5A, which may be due to a part of particles aggregated during the freeze-drying process. TEM images (Figure 5A) showed that the AgNPs were evenly dispersed in solution, the particle sizes (Figure 5B) were about 5–15 nm and the shapes were round. The results confirmed that Lceo as a natural essential oil obtained enormous potential in green-synthesis of AgNPs with small particles and great dispersion adventures.

#### 5.2.4 XRD test

**FIGURE 6:**
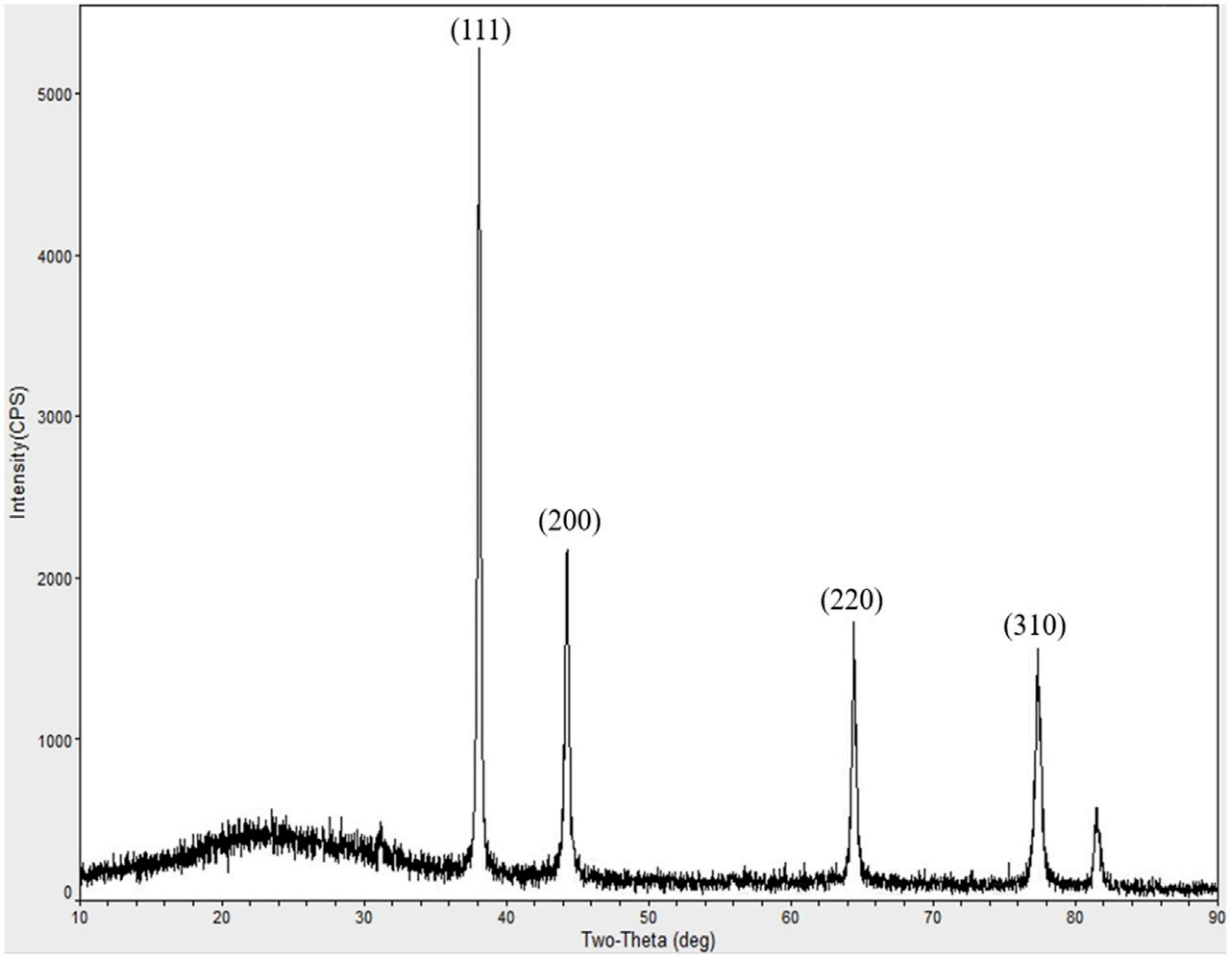
XRD analysis spectrum of Lceo-AgNPs.

The XRD patterns of Lceo-AgNPs (Figure 6) show four diffraction peaks corresponding to (110), (200), (220), and (310) at 2θ = 38.10°, 44.17°, 64.26°, and 77.42°, respectively, further proving the generation of Lceo-AgNPs. In addition, the presence of Ag elements in the form of face-centered cubic lattice is also shown.

### 5.3 Antibacterial effect of Lceo-AgNPs against *EC 14* and *MRSA*

#### 5.3.1 Multidrug-resistant assay

The widespread usage of antibiotics promoted the development and rapid spread of bacterial resistance, heralding decreasing antibiotic potency in humans and animals. The MIC results of antibiotics for *E. coli* are shown in Table 1. As shown in Table 2, *EC14* was resistant to seven antibiotics including rifampicin, tetracycline, colistin, fossils, penicillin, amikacin, ampicillin, and intermediate with meropenem, which determined the broad spectrum and strong biotic-resistance of *EC 14*. In this case, *EC14* can be considered *MDR,* which is defined as strains that are resistant or insensitive to at least three different classes of antimicrobials (Pungle Rohini et al., 2021). Otherwise, the MIC of Lceo-AgNPs on *EC 14* and *MRSA* was 25 μg/ml and the MBC was 50 μg/ml.

**TABLE 1:** MIC and antimicrobial susceptibility results of *E. coli*.

Antibiotics (5,120 μg/ml)	*EC 14*	
	MIC (μg/ml)	Antimicrobial susceptibility
Rifampicin	512	R
Tetracycline	256	R
Meropenem	2	I
Colistin	8	R
Fossils	512	R
Penicillin	512	R
Amikacin	512	R
Ampicillin	512	R

**TABLE 2:** Wound areas of mice in each group.

Group	Wound areas (mm^2^)				
	0 d	1 d	4 d	7 d	14 d
**B**	64	42	20	9	9
	81	35	32	21	21
	64	42	30	25	25
**C**	56	31	27	19	0
	96	42	25	19	2
	64	36	29	11	0
**D**	64	48	45	1	0
	58	44	25	3	4
	58	39	21	0	0

The outcomes of this work revealed that the green-synthesized Lceo-AgNPs nanoparticles played a role in excellent antibacterial activity which can serve as a great bactericidal agent.

#### 5.3.2 Results of time-killing curve test

**FIGURE 7:**
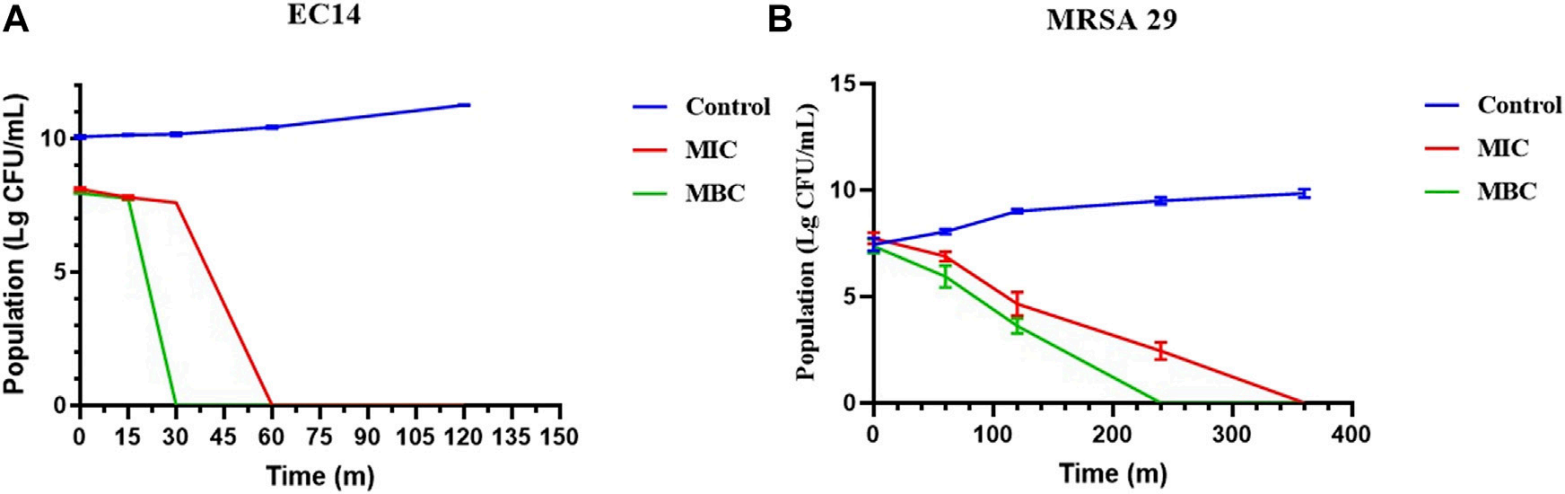
Time-killing curves of *E. coli* **(A)** and *MRSA* **(B)** treated with Lceo-AgNPs at the concentrations of MIC and MBC.

The time-killing curves were implemented to detect the bactericidal efficiency of Lceo-AgNPs. As shown in Figure 7A, compared with the control, MIC and MBC of Lceo-AgNPs played bactericidal roles as soon as being attached to the *E. coli* at 0 min. As for MIC of Lceo-AgNPs, during the first 15 min, the germicidal rates of MIC and MBC groups were similar. After 15 min, the germicidal rate of the MBC group significantly increased and the number of *EC14* decreased exponentially. However, the MIC group played a significant role in killing bacteria at 30 min. For MIC and MBC groups, no bacteria were discovered at 30 and 60 min, respectively. As shown in Figure 7B, Lceo-AgNPs in MIC could kill *MRSA* after 6 h while MBC Lceo-AgNPs play a killing effect after 4 h. These results are consistent with scientific evidence of bacterial structure.

#### 5.3.3 SEM result

**FIGURE 8:**
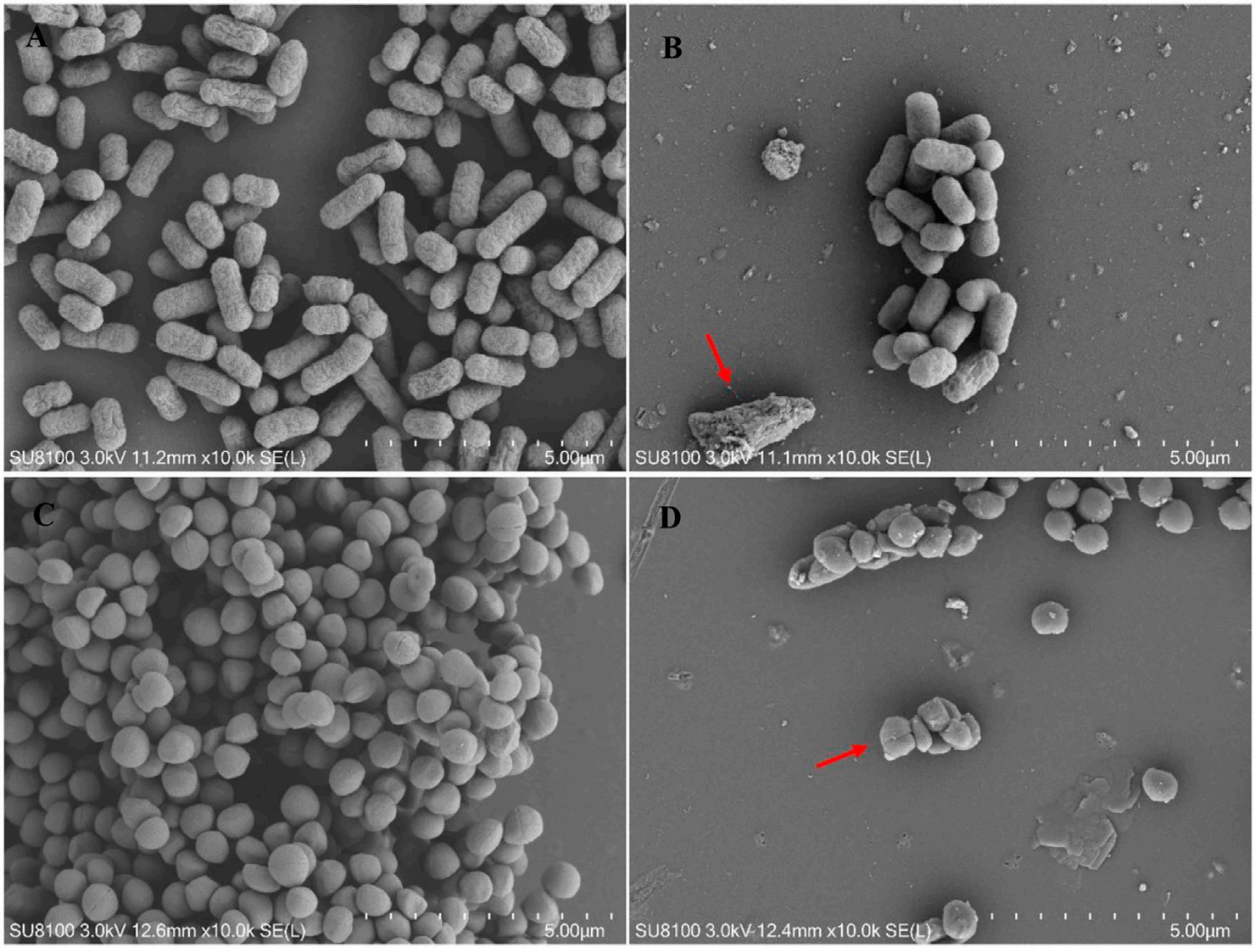
SEM images of *E. coli* **(A,C)** and *MRSA* **(B,D)** treated with/without Lceo-AgNPs. **(A,C)** negative control magnified ×10,000; **(B,D)** AgNPs-treated bacteria magnified ×10,000.

Changes in the morphology of bacterial cells treated with Lceo-AgNPs are exhibited through SEM images. The images of controlled *E. coli* and *MRSA* without treatment of Lceo-AgNPs stayed in natural form with clear cell boundary as shown in Figures 8A,C, respectively. Compared with the controls, the quantities of bacterial cells were considerably reduced and the normal cell morphology of *E. coli* treated with Lceo-AgNPs vanished, the cells were deformed and broken and the residues of rupturing *E. coli* cells clustered as marked with red arrows (Figure 8B). Some bacterial cells began to atrophy and lost natural morphology, became unsaturated. Bacterial lysis was noticeably observed as marked with red arrows (Figure 8D). These results indicated that Lceo-AgNPs were able to disrupt the structure of the bacteria and thus acted as a bactericide.

### 5.4 Cytotoxicity assay

**FIGURE 9:**
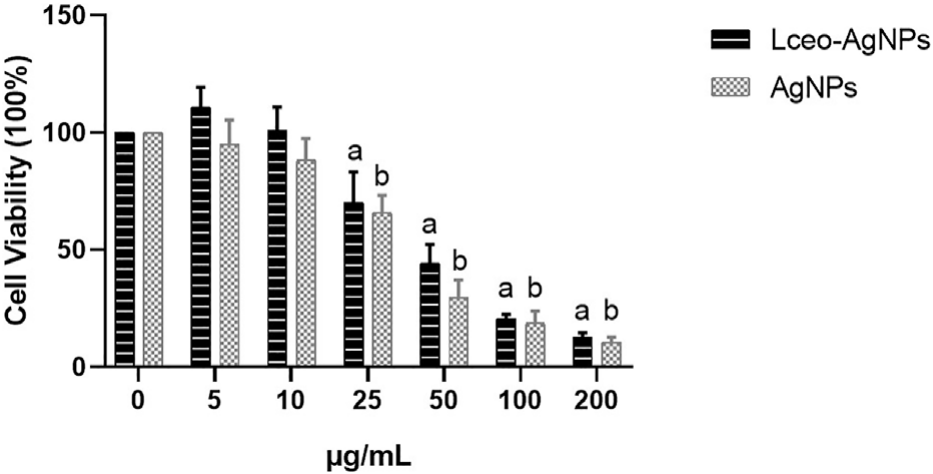
Cytotoxicity of Lceo-AgNPs and AgNPs in macrophages. 0 μg/ml as control group and the data were analyzed using *t*-test to determine significance of difference. a: significant difference when the Lceo-AgNPs-treated group were compared to control group; b: significant difference when the AgNPs-treated group were compared to the control group.

Cell viability was executed through the MTT assay utilizing different concentrations of AgNPs in macrophages after 24 h of treatment. As shown in Figure 9, 25 μg/ml and higher concentrations of Lceo-AgNPs and AgNPs exhibited more cell toxicity. Compared with AgNPs, Lceo-AgNPs showed smaller cytotoxicity. The rate of cell viability of macrophages disposed with 5, 10, 25, 50, 100, and 200 μg/ml Lceo-AgNPs were 111.09%, 134.87%, 70.24%, 44.24%, 20.45%, and 12.40%, respectively. The rates of cell viability of macrophages disposed with 5, 10, 25, 50, 100, and 200 μg/ml AgNPs treatment were 95.02%, 88.37%, 65.78%, 29.64%, 13.37%, and 10.47%, respectively. In addition, Lceo-AgNPs with low concentrations had an effect on promoting proliferation of macrophages. This result implied that Lceo-AgNPs in the green-synthesis route could decrease cytotoxicity.

### 5.5 *In vivo* evaluations

#### 5.5.1 Treatment of infected wounds and healing rate

**FIGURE 10:**
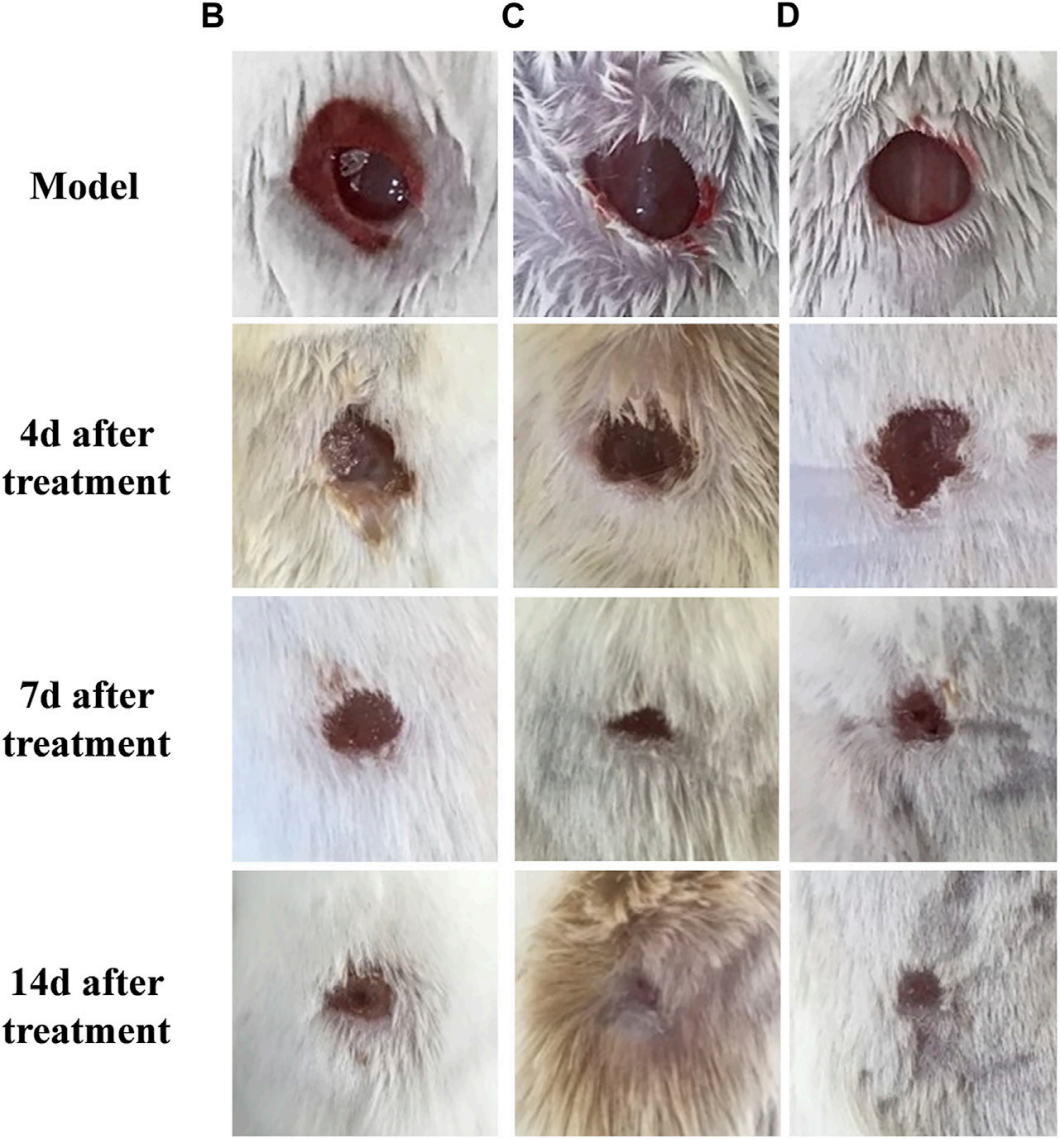
Photographs of skin wounds at 4, 7, and 14 days after treatment.

The mice in groups B, C, and D were depressed, less active, and had less food and water intake. The general observation results of mice with different treatments are shown in Figure 10. After setting up infection wound model for 24 h, exudate and hemorrhage appeared on the whole wounds. On the fourth day of treatment, the wound surface was moist with exudate and hemorrhage and no fresh granulation tissue generation was evident in group B. By contrast, there was a large amount of fresh granulation tissues in the wound without obvious exudate in group C, which indicated Lceo-AgNPs possessed inhibitory effect on *MRSA* infection. A small amount of epithelial growth was visible at the margin of group D. On the seventh day of healing, a layer of scab was formed over the wound in group B while the wound areas of group C and group D significantly diminished. During the 14th day, the wounds of group C and group D were completely healed, but the wounds of group B were still scabby and not completely healed.

**FIGURE 11:**
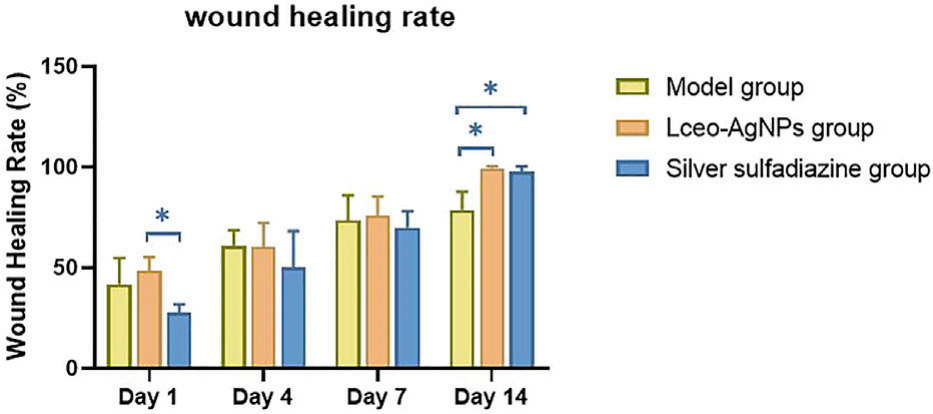
Wound-healing rate at different timepoints including days 1, 4, 7, and 14 after different treatments (*p < 0.05).

The wound area of each group of mice is shown in Table 2, and the wound-healing rate was calculated based on the wound area and shown in Figure 11. Group C showed a significant promotion in wound healing of 48.4% on days 1 in comparison with 24.5% of group D (*p* < 0.05). Moreover, group C and group D showed a significant promoting effect on wound healing of 100% compared with 78.7% of group B.

#### 5.5.2 Result of H&E stain

**FIGURE 12:**
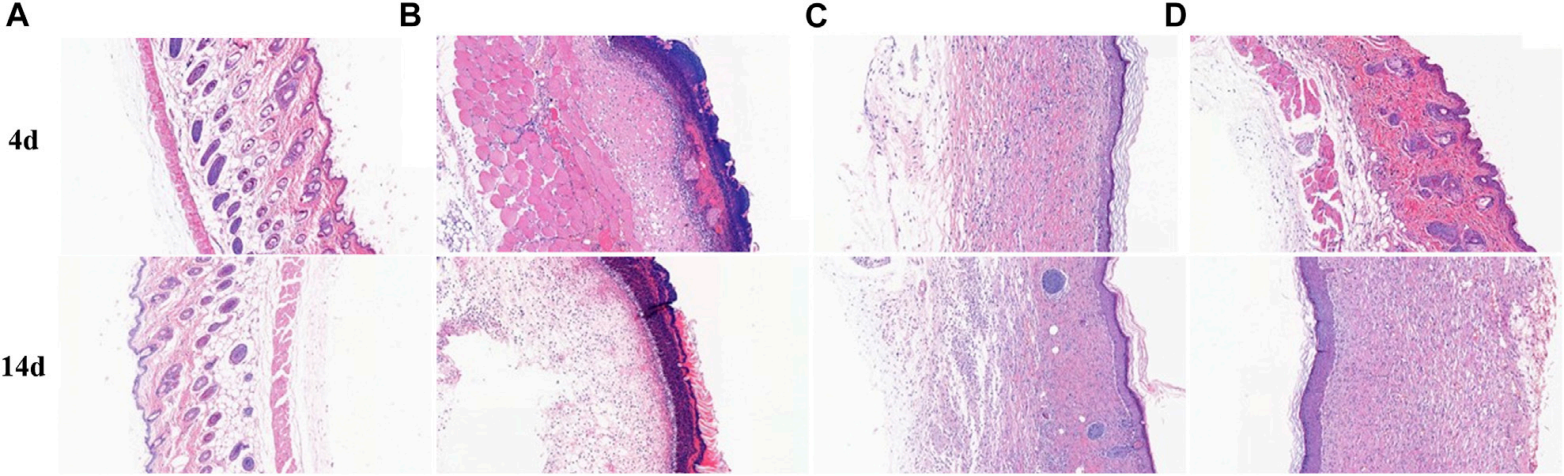
H&E staining of healthy and wound skin sections after 4 and 14 days with different treatments.

Histopathology observation of wound tissue was conducted to assess healing activity with different treatments. As shown in Figure 12, 4 days after treatment, focal incomplete keratinization in which homogeneous, red-stained material (plasma), and inflammatory cells (crusting) are visible, necrotic tissues and congested tissue underlying abscesses were visible in group B. The inflammatory cells distributed in wound tissue were mainly neutrophils. Compared with group B, wound tissues of group C exhibited thickened epithelium and vigorous growth of granulation tissue. The epidermal spine layer showed mild hyperplasia, the dermal papillae became edematous, and the collagen fibers showed thickening and red staining. There was a moderate density of mixed inflammatory cells infiltrating the epithelium around blood vessels. There were slight bleeding and amounts of immature fibroblasts in wound skin. There was no obvious epithelial tissue found in group D, while a lot of congestion filled in the tissue. After being treated for 14 days, there was no obvious new epithelium in group D, but the tissue was still hyperemic in group B. In group C and group D, intact thickened epithelium and few inflammatory cells were present in wounds. Furthermore, the congestion vanished.

#### 5.5.3 Result of ELISA

The wound-healing effects of Lceo-AgNPs were estimated from the ELISA data illustrated in Table 3. After successful modeling, the CPR and type I collagen content in group B differed significantly compared with group A indicating that the mice developed an inflammatory response and lost skin collagen after modeling. After 4 days of treatment, the CPR and type I collagen contents of groups B, C, and D were significantly different compared with group A, indicating that the inflammatory reaction still existed and the collagen content was low. Among them, the content of CPR and type I collagen in groups C and D differed significantly compared with group B, indicating that Lceo-AgNPs and silver sulfadiazine have some effect on relieving inflammatory symptoms as well as promoting collagen production. After 14 days of treatment, the differences in CPR and type I collagen content in group B compared with group A were significant, while the differences in CPR and type I collagen content in group C and type I collagen content in group D compared with group A were not significant, indicating that Lceo-AgNPs had strong anti-inflammatory effects as well as the ability to promote collagen production and wound healing, and silver sulfadiazine had a better ability to promote wound healing. Lceo-AgNPs had a stronger ability to inhibit the inflammatory response compared to silver sulfadiazine.

**TABLE 3:** CRP and I collagen results are presented as SD ± SE; statistically significant data are given as^a,b,c,d^ *p* < 0.05.

Indexes	Groups			
	A		A	
0th day after administration				
CRP	1304.41 ± 59.68^b^	1428.66 ± 7.17^a^		
I collagen	419.14 ± 4.72^a^	366.47 ± 3.31^b^		
4th day after administration				
CRP	1353.39 ± 23.50^c^	1634.13 ± 7.46^a^	1589.93 ± 22.38^b^	1605.46 ± 30.48^b^
I collagen	418.06 ± 12.76^a^	321.01 ± 7.68^c^	345.19 ± 8.19^b^	321.38 ± 12.64^c^
14th day after administration				
CRP	1447.77 ± 39.31^c^	1626.97 ± 26.89^a^	1361.76 ± 29.19^c^	1556.48 ± 20.69^b^
I collagen	418.78 ± 9.46^a^	318.49 ± 6.80^b^	410.84 ± 17.40^a^	377.29 ± 22.22^a^

## 6 Discussion

Lceo mainly comes from fruit, and the essential oil separated and purified from this part contains the most kinds of chemical elements, which can inhibit the growth of microorganisms in food, and has natural safe, non-toxic, and benign characteristics. The main chemical components of Lceo are aldehydes, ketones, esters, and nitrogenous. Although China is the largest country in the cultivation of *L. cubeba*, due to its low production level, the export of foreign trade is mainly principal crude oil, which belongs to the workshop mode of high cost and low output, so it cannot realize the advantage in utilization of this resource (Yang et al., 2018; Wang et al., 2019).

To pursue safer cure circumstances, there are many simple, fast, and effective methods for preparing AgNPs. Interestingly, most green-synthesis materials are plant extracts, but the preparation of AgNPs by using essential oil as a reducing agent is scarce. As a natural safe plant extractive, Lceo has effects on avoidance, insecticide, antioxidants, and other bioactivities, which can be utilized in multiple fields including spice synthesis, food additives, medical care, biological treatment and food preservation, and possessed high economic values (Li et al., 2007). On the contrary, the applications of Lceo are limited duo to its poor water solubility and thermal stability, volatilization, easy oxidation, and deterioration during storage. To avoid these weaknesses, exploiting novelty, efficient, and safe products of Lceo has been considered as a new point of penetration.

### 6.1 Synthesis and characterization of Lceo-AgNPs

The synthesis process of AgNPs is accomplished in two steps: firstly, Ag^+^ is reduced to Ag^0^ in the presence of biological catalysts. Then, oligomeric clusters are formed through agglomeration and Ag^0^ is led to silver particles (Mashwani et al., 2016). Bakkali F. considered low molecular weight aromatic and aliphatic components, like terpenes derived from essential oil, played a positive effect on synthesis of AgNPs (Bakkali et al., 2008). In a separate study, it has been proposed that the key mechanism behind plant-mediated nanosilver synthesis was plant-assisted reduction caused by phytochemicals. The leading phytochemicals contained ketones, terpenes, amides, flavonoids, carboxylic acids, and aldehydes (Pohlit et al., 2011). Water-soluble phytochemicals including organic acids quinones and flavones can instantaneously restore the silver ions in the reactive mixture (Doughari, 2012). Those strongly proved the great potential and development ability of essential oils in the green synthesis of silver nanoparticles. The polar hydroxyl and aldehyde groups in Lceo are more likely to lose protons and acquire negative charge, while the Ag^+^ is considerably bound through electrostatic interaction, which is also conducive to the reduction reaction and enhancing reduction rate.

According to a previous study (Prathna et al., 2011), AgNPs formation was verified through UV-vis. Silver possessed unique optical properties due to the SPR, which was the collective oscillation phenomena of conduction electrons. According to other studies, synthetic AgNPs appeared brown because of the surface plasmon vibration excitation, which also had a maximum absorbance peak on 420–450 nm (Saravanakumar et al., 2021). This was definitely consistent with the results of this study. When the content of Lceo progressively increased, the number of Lceo-AgNPs also increased, and the silver nanoparticles were in the formation state, with a gradually increasing number and comparatively small particle size. When the ratio of reducing agent to silver nitrate reaches 3:1, Ag^+^ could be completely reduced to silver nanoparticles. At this time, the silver nanoparticles were in the nucleation stage and the number of silver nanoparticles increased. When the ratio exceeded 3:1, the reducing agent was insufficient to restore all Ag^+^. At this time, the reaction rate and efficiency decreased, silver nanoparticles began to aggregate, and the number decreased. According to previous studies, the shape and size of AgNPs could be chosen through different reaction properties used for the synthesis (Mohanty et al., 2012).

To obtain silver nanoparticles, several analytical techniques were utilized, including UV-vis, TEM, XRD, zeta potential, and FTIR. In general, the sizes of AgNPs in green-route were larger compared with the particles synthesized by chemical methods (Choi et al., 2021). Priyanka Basera reported that AgNPs prepared by *Cymbopogon citratus* were about 35 nm (Khan et al., 2017). Yasmin M. Heikal et al. reported that the size of AgNPs synthesized by aqueous leaf extract of *Eichhornia crassipes* was about 56–58 nm (Mohanta et al., 2017). The AgNPs produced by *Psidium guajava* L. leaf aqueous extracts were approximately 20–35 nm as reported (Pugazhendhi et al., 2015). Renuka Yadav et al. utilized *Litsea longum* L. extract to generate AgNPs at 28.8 nm in a green-synthesis way (Kirkwood and Melov, 2011). Most results of AgNPs sizes prepared in green synthesis ways were higher than the result in this study. The Lceo-AgNPs particle size exhibited by DLS was different from TEM results, which may be caused by the presence of hydration-capping agent and solvation effect (Mukherjee et al., 2008). In addition, TEM measured the particle size of the solid sample, while DLS measured the particle size of the aqueous sample. The hydrodynamic diameter of nanoparticles in solution is larger than the data measured by TEM (She et al., 2013). As previous research shows, factors such as the particle size of AgNPs directly modified its impacts. In order to guarantee the resistance effects of AgNPs on bacteria, cancer, and other microbial diseases, it is imperative to pay attention to its properties and stability. The silver nanoparticle size and distribution strongly depend on biological compounds in plant extracts. Reducing agents of extracts promote quick reaction rate, which can be conducive to the formation of smaller nanoparticles. When decreasing the overall surface energy, individual nanoparticles interact with each other *via* chemical bonds and physical attraction forces at interfaces, resulting in agglomeration. Here emphasizes the significance of stabilizer (Maillard et al., 2018). In this study, the natural chemical agents were attached to the surface of Lceo-AgNPs as capping agent and stabilizer to inhibit aggregation and decrease cytotoxicity.

The stability of Lceo-AgNPs could also be intuitively reflected by zeta potential. The zeta potential is an important physicochemical property that affects antibacterial activity of AgNPs because the interaction between nanoparticles and cell membranes is based on electrostatic adhesion (Mandal et al., 2016). The negative chemical bonds in biological compounds will attract each other with positive AgNPs, thus acting as stabilizers. The significance of the zeta potential is that its value is related to the stability of colloidal dispersion. The zeta potential is a measure of the strength of the mutual repulsion or attraction between particles. The smaller the molecule or dispersed particle, the higher the zeta potential (positive or negative), the more stable the system, dissolution or dispersion can resist aggregation. The lower zeta potential is, the more easily silver nanoparticles condense. The negative value of particle surface charge could well explain the dispersion and stability of AgNPs due to particle repulsion. The negatively charged AgNPs synthesized in green-route interacted with the membrane through electrostatic attraction with the polar heads of the lipids, which displayed higher bactericidal effect than AgNPs synthesized by citrate. This was because of the hydrophobic effect of capping agents during the process of green-route synthesis, revealing the strong advantages of green chemistry in antibacterial activity of AgNPs (Roy et al., 2019). The AgNPs synthesized through green-route using Lceo were stable and had significant dispersion to maintain biochemistry activity, which may be due to the biomolecules attached to the surface of Lceo-AgNPs significantly increased surface charge of Lceo-AgNPs, thereby enhancing stability through inhibiting Lceo-AgNPs aggregation. FTIR results confirmed the above inference: Lceo provided chemical bonds on the surface of silver nanoparticles, including C=C, N-O, C-C, C=O, H-C-H, and O-H, which acted as stabilizers. The citral, limonene, linalyl isobutyrate, α-Vinyl acetate, geraniol, and other organic substances in Lceo endow nano-silver with rich functional groups, which enable Lceo to play the role of a stabilizer.

### 6.2 Bactericidal effect of Lceo-AgNPs

The current COVID-19 pandemic is a wake-up call to seriously rethink our preparedness to deal with infections (Aghila Rani et al., 2021). The groundbreaking discovery of antibiotics has revolutionized modern medicine and saved countless lives. Nevertheless, our current arsenal of effective antibiotics is rapidly being depleted due to the spread of MDR bacteria (Bragginton and Piddock, 2014; Hamad et al., 2019). Antibiotic-resistant bacteria induced by overuse of antibiotics has been a major public health problem (Naik et al., 2000; Kirkwood and Melov, 2011). In this context, infection prevention and control are critical to prevent the spread of multidrug-resistant bacteria. *E. coli* as a common pathogen of bacterial diseases can incorporate drug-resistant genes into other bacteria by means of horizontal transmission and vertical transmission, thus creating more drug-resistant strains (Moritz et al., 2020; Le Guern et al., 2021). The resistance mechanism of *MRSA* is that the binding protein has a very low affinity with antibacterial drugs, which can promote the synthesis of bacterial cell wall. Therefore, β-Lactam antibacterial drugs cannot obstruct the synthesis of cell wall peptidoglycan layer, resulting in drug resistance (Sansom et al., 2021). The drug resistance rate of penicillin, cephalosporin, erythromycin, azithromycin, and other conventional antibacterial drugs is increasing, and some have even attained 80%–90%. Meanwhile, the development of new antimicrobials has been slow (Hu et al., 2020). AgNPs, which are less likely to develop drug resistance, can resolve this problem by applying it to the treatment of multidrug-resistant bacteria.

This research studied antimicrobial activity of Lceo-AgNPs against antibiotic-resistant *E. coli* and *MRSA*. The MIC and MBC of *EC14* and *MRSA* were 25 and 50 μg/ml. This research indicated that the Lceo-AgNPs possessed strong bactericidal effects and killed G^−^ and G^+^ bacteria within 2 and 6 h, respectively. Gram-positive bacteria have thicker cell walls than gram-negative bacteria that make them more resistant to the environment, which explains why Lceo-AgNPs require more time to kill gram-positive bacteria (Moraes et al., 2020). The SEM result discovered Lceo-AgNPs damaged bacteria form and the dead bacteria residue huddled together. The interaction and adhesion of AgNPs with bacterial surface could modify cell wall structure, induce intracellular production of reactive oxygen species (ROS), disrupt plasma membrane, alter protein interactions, obstruct DNA replication, and damage the macromolecules (lipids, proteins, DNA, and RNA) (Patra and Baek, 2017; El-Faham et al., 2020). Up to now, AgNPs have been unable to induce resistance in any bacterial strains tested, which have been approved by the US Food and Drug Administration (FDA) as a fungicide for burn wound dressings (Mao et al., 2016). In this way, developing new-type pharmaceutical preparations using Lceo-AgNPs to apply to clinical treatment is a feasible method (Roy et al., 2012).

### 6.3 Cytotoxicity of AgNPs

Trauma refers to all skin lesions or disorders resulting from trauma or treatment conditions. The morphological features and functions will be impacted once the trauma forms. There is a high possibility of microbials, especially pathogenic bacteria, invading the wound, which may alleviate the healing process (Zare et al., 2021). Fibroblast as an essential combination in the wound-healing process can synthesize and secrete skin collagen which will fill damaged tissues and promote epithelial regeneration (Devanesan and AlSalhi, 2021). Macrophages belong to immune cells, which perform essential roles in nonspecific immunity of the body and inflammation process. Hence, the cytotoxicity assay of Lceo-AgNPs and silver nanoparticles synthesized through chemical method on macrophages implied the cytotoxic impact of Lceo-AgNPs in green-synthesis way obviously reduced, which protected the immune ability of inflammatory response in mice. This is consistent with the results reported by Jena et al. (2012) who demonstrated nano-silver in sterilization doses of macrophages have no obvious poisonous characteristics to the cells or DNA damage. Lee et al. (2021) compared bare AgNPs with coated AgNPs. As the results showed, the toxicity of AgNPs was decreased through coating on the surface of silver nanoparticles. Different from chemical methods, the reduction and stabilization of green synthetic silver nanoparticles are achieved through biocompatible materials, and hence the toxicity problem is alleviated (Cao et al., 2021).

### 6.4 Glycerin–gelation ointment

Sulfadiazine silver cream and sulfadiazine silver sulfadiazine zinc cream are commonly used as external anti-infection agents for burns and scalds. Nevertheless, after transdermal absorption of sulfadiazine silver, systemic adverse reactions of sulfadiazine drugs can occur, such as anaphylaxis, neutropenia or deficiency, thrombocytopenia, and aplastic anemia. There is an urgent need to develop new pharmaceutical preparations with high safety and antibacterial spectrum to replace sulfadiazine silver cream. Glycerin–gelatin could be prepared into water-soluble suppository, emulsion matrix, and hydrogel (Cassano et al., 2020). Glycerin can be utilized as a lubricating oil to moisturize the skin. Gelatin is made from animal skin and bone, which is a denatured product of collagen filled with enriched protein (Nadim et al., 2017). It is a food-grade hydrolysate of collagen, which can steadily absorb discharge of wound and soften it. Previous studies have shown that glycerin and gelatin could be blended in a certain proportion to prepare a transparent paste, which could dissolve in secretions and prolong the curative effect when mixed with drug solutions. Glycerin can keep suppositories from drying out, and the higher the concentration, the more readily the drug dissolves. Glycerin–gelatin suppository owns characteristics of being elastic, not easy to crack, could be gradually dissolved in secretions and prolong the curative effect of drugs (Chen et al., 2011). Otherwise, the more content of glycerin and water is, the easier to dissolve the suppository. In general, the best ratio of water, gelatin, and glycerin is 10:20:70. The previous study has shown that hydrogels could be made from glycerin, gelatin, and water (Sanwlani et al., 2011; Amiri et al., 2020). Nevertheless, the preparation process of injectable hydrogels is strict, the higher requirements and standards on gelling temperature and acid base of hydrogels need to be satisfied in clinical application (Ahovan et al., 2020). The suppository with more inexpensive and handy characteristics can overcome the shortcomings of hydrogel and be deployed to treat skin-infected wounds in the most common skin drug delivery way (Cheng et al., 2018). In consideration of clinical practicability and new drug development, we adopted glycerin–gelatin loaded with Lceo-AgNPs to prepare primary Lceo-AgNPs ointment.

### 6.5 *In vivo* study

Although we are hopeful about the antibacterial effect of silver nanoparticles *in vitro*, the adverse effects of those on humans and animals are our concern about the use of AgNPs. Therefore, we evaluated the antibacterial effect of AgNPs using a live animal model. Rapid and efficient wound recovery could alleviate discomfort and prevent infection, particularly inhibiting scar formation caused by excessive synthesis of extracellular matrix proteins (Gao et al., 2020). *In vivo* assay discovered Lceo-AgNPs treatment definitely promoted *MRSA*-infected wound healing. After being treated for 14 days, the mice wounds with Lceo-AgNPs treatment and sulfadiazine silver treatment groups had already healed, while the wounds without any treatment had not recovered. CRP plays an active role in inflammatory response and makes body resistant to non-specific diseases. As an acute phase protein, it rapidly increases after the onset of inflammation and then decreases to normal when it improves. Type I collagen is the most abundant collagen in the body and can form collagen fibers, which exists in scar tissue and promotes wound healing (Yamaguchi et al., 2018). Compared with model group, CRP index of mice treated with Lceo-AgNPs decreased, indicating that Lceo-AgNPs promoted the disappearance of inflammatory response. Zhou et al. (2021) discovered that the expression of proinflammatory factors (IL-6 and IL-1β) was significantly downregulated while the growth/differentiation factor (TGF-β) and vascular endothelial cells were significantly upregulated after AgNPs treatment. Adeyemi and Adewumi (2014) reported alanine transaminase (ALT) and aspartate transaminase (AST) levels in serum and tissues of rats were significantly increased after AgNPs treatment. Nano-silver also promotes granulation tissue formation and maturation in the early stages of wound healing by reducing inflammatory cytokines such as nitric oxide and prostaglandin E2 (Diniz et al., 2020). In addition, the significantly increased content of type I collagen also demonstrated that Lceo-AgNPs can promote wound healing. Glycerin–gelatin suppository loaded with Lceo-AgNPs played a bactericidal role through providing a nano-physical barrier with wound-healing activity and releasing Lceo-AgNPs. The glycerin–gelatin covered-infected wounds can act as a physical barrier to keep pathogenic bacteria out, while also transporting antibacterial active ingredients (Kalantari et al., 2019). In this way, Lceo-AgNPs suppository could be developed into new-type drugs to substitute traditional antibiotics like silver sulfadiazine.

### 6.6 Limitations and perspectives

This research explored the green synthesis of Lceo-AgNPs and confirmed that the synthesized Lceo-AgNPs had the advantages of small particle size, great stability, and better dispersion, and the synthesis process was simple and rapid. In addition, Lceo-AgNPs had strong bactericidal effect on drug-resistant bacteria, and the *in vivo* experiments further confirmed the potential of Lceo-AgNPs to replace antibiotics for clinical treatment. The above experiments studied on macroscopic aspects, and there was a lack of microscopic mechanism exploration to study the bactericidal mechanism of Lceo-AgNPs against drug-resistant bacteria and the mechanism of promoting wound healing. The combination of macroscopic and microscopic studies would lead to the application of Lceo-AgNPs as an alternative to antibiotics in clinical treatment and reduce the generation and development of antibiotic resistance in a real sense.

## 7 Conclusion

In conclusion, we first discovered a novel way for the rapid, valid, eco-friendly, and safe synthesis of AgNPs using Lceo as a reductant and capping agent. The Lceo-AgNPs had a maximum absorption peak of UV-vis at 423 nm and the size of the AgNPs was about 8–15 nm according to TEM results. According to the result of FTIR, the stability of Lceo-AgNPs was increased due to C-H, C=C or N-O, -C-C, C=O, H-C-H, O-H, and other organic compounds like alkyne, aromatic hydrocarbon attached to Lceo-AgNPs, which was confirmed by high zeta potential. Lceo-AgNPs exhibited positive antibacterial activity against multidrug-resistant *E. coli* and *MRSA*. In addition, Lceo-AgNPs with low concentrations exhibited promoting proliferation of macrophages while Lceo-AgNPs below 25 μg/ml possessed no significant cytotoxicity on macrophages. As for *in vivo* experiment, Lceo-AgNPs could promote *MRSA*-infected wound healing. These results indicated that Lceo as a natural medicine had the potential of preparing AgNPs in a green-synthesis way which had functions of bactericidal activity against microbials like *E. coli* and *MRSA*. Based on these results, Lceo-AgNPs could replace antibiotics and decrease the development of bacterial resistance, which would be a breakthrough in the treatment of bacterial infection. This study could make basics in pharmaceutical preparation and clinical research, as well as be beneficial in the formulation of bactericidal products.

This study adopted a green-synthesis way to prepare Lceo-AgNPs with small particles, great stability, and bacteriostatic activity against multidrug-resistant *E. coli* and *MRSA*. The glycerin–gelatin suppository loaded with Lceo-AgNPs broke strict preparation process and limitations of clinical application of hydrogel, accelerated fibroblast proliferation, collagen synthesis, and angiogenesis of *MRSA*-infected wound, and then facilitated the early tissue granulation formation and re-epithelialization of full-layer skin wound and promoted wound healing. This study opens up the new clinical application road of Lceo, and lays the foundation for development of clinical agents of Lceo-AgNPs.

## Data Availability

The original contributions presented in the study are included in the article/supplementary material, and further inquiries can be directed to the corresponding authors.
